# Microbiome and Human Aging: Probiotic and Prebiotic Potentials in Longevity, Skin Health and Cellular Senescence

**DOI:** 10.3390/nu13124550

**Published:** 2021-12-18

**Authors:** Jacqueline Lena Boyajian, Merry Ghebretatios, Sabrina Schaly, Paromita Islam, Satya Prakash

**Affiliations:** Biomedical Technology and Cell Therapy Research Laboratory, Department of Biomedical Engineering, Faculty of Medicine, McGill University, 3775 University Street, Montreal, QC H3A 2B4, Canada; jacqueline.boyajian@mail.mcgill.ca (J.L.B.); merry.ghebretatios@mail.mcgill.ca (M.G.); sabrina.schaly@mail.mcgill.ca (S.S.); paromita.islam@mail.mcgill.ca (P.I.)

**Keywords:** cellular senescence, gut microbiome, skin, disease, dysbiosis, microbial metabolites, nutrition, prebiotics, probiotics

## Abstract

The role of the microbiome in human aging is important: the microbiome directly impacts aging through the gastrointestinal system. However, the microbial impact on skin has yet to be fully understood. For example, cellular senescence is an intrinsic aging process that has been recently associated with microbial imbalance. With age, cells become senescent in response to stress wherein they undergo irreversible growth arrest while maintaining high metabolic activity. An accumulation of senescent cells has been linked to various aging and chronic pathologies due to an overexpression of the senescence-associated secretory phenotype (SASP) comprised of proinflammatory cytokines, chemokines, growth factors, proteases, lipids and extracellular matrix components. In particular, dermatological disorders may be promoted by senescence as the skin is a common site of accumulation. The gut microbiota influences cellular senescence and skin disruption through the gut-skin axis and secretion of microbial metabolites. Metabolomics can be used to identify and quantify metabolites involved in senescence. Moreover, novel anti-senescent therapeutics are warranted given the poor safety profiles of current pharmaceutical drugs. Probiotics and prebiotics may be effective alternatives, considering the relationship between the microbiome and healthy aging. However, further research on gut composition under a senescent status is needed to develop immunomodulatory therapies.

## 1. Introduction

**Figure 1:**
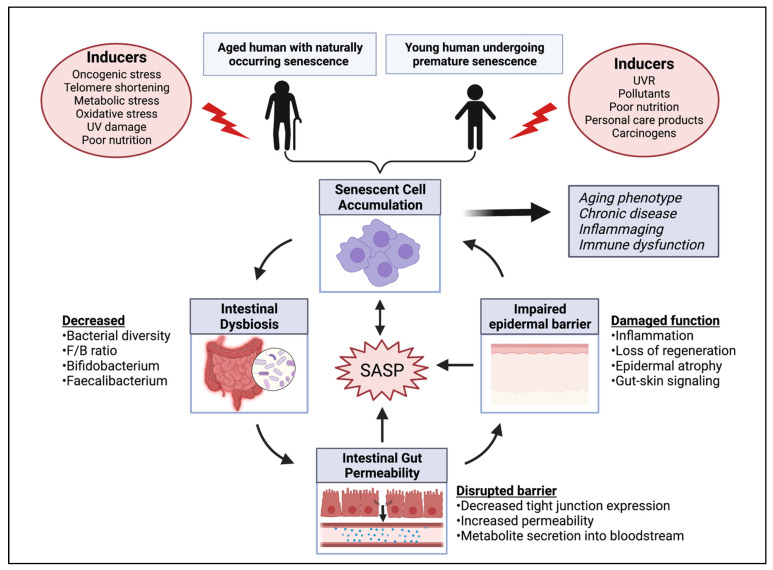
Gut microbiome and its impact on gut dysbiosis, aging and skin health. The consequences of senescent cell accumulation including gut microbial imbalance, subsequent increased gut barrier permeability, secretion of microbial metabolites and a resulting impairment to the skin. The senescence associated secretory phenotype (SASP) remains central to each of the factors presented. The pathogenesis of age-related and chronic diseases, systemic inflammation and immune decline results from the proposed interrelationship. UV = ultraviolet; UVR = ultraviolet radiation; F/B = Firmicutes/Bacteroidetes. Created with Biorender.com.

The global phenomenon of population aging has prompted an ongoing discussion of anti-aging targets. The literature has developed a strong association between a balanced gut microbiome and healthy aging. However, an intrinsic hallmark of aging, known as cellular senescence, is only beginning to be investigated in relation to the gut. Although senescent cells are beneficial when they present acutely, an accumulation of these cells can lead to deleterious effects on the tissue environment through secreted factors known as the senescence-associated secretory phenotype (SASP). Such signaling pathways promote inflammaging and tissue dysfunction, thereby playing a putative role in systemic metabolic dysfunction and various age-related pathologies. This review intends to propose an interrelationship between microbial dysbiosis, senescence and systemic health (Figure 1). In particular, skin health will be emphasized based on findings regarding its impact on healthy aging, the gut-skin crosstalk and senescent cell accumulation within the skin. This review also aims to extend the present discussion of senescence by presenting recent findings that link senescent cells to several disorders of the integumentary system including skin carcinogenesis, psoriasis, wound healing and skin barrier immunity, among others. Additionally, a framework will be provided that connects various skin diseases to the presence of senescent cells through SASP associated pathways. The use of omics techniques, particularly metabolomics, is proposed as a method to characterize metabolites associated with senescence and their impact further. Associations drawn between the gut microbiome, aging and systemic impact emphasize the need for effective anti-senescent therapeutics. Current senolytic and senostatic therapies are outlined, including pharmaceutical agents and their limitations. The anti-senescent potential of nutraceutical compounds as well as probiotics and prebiotics will also be examined. Lastly, industry efforts made to adopt a senescent-forward approach for consumer health and longevity will be highlighted. Overall, the potential of targeting cellular senescence for anti-aging and systemic health is discussed based on recent findings.

## 2. Gut Microbiome and Human Aging

The gastrointestinal (GI) (GIT) tract harbors a complex ecosystem that is made up of trillions of bacteria, fungi and microorganisms, known as the gut microbiota [1]. The gut microbiome works to maintain host health and homeostasis through a delicate balance of commensal and pathogenic bacteria. Microbial communities within the human GI aid in the production and synthesis of vitamins, amino acids, SCFAs and metabolites through enzymatic activity and metabolic pathways [2]. Together, these contribute to food digestion, xenobiotic metabolism and the assembly of various bioactive molecules. A disruption to the intestinal microflora can result in microbial dysbiosis. A dysbiotic state is characterized as a reduction in bacterial species diversity as well as the loss of beneficial bacteria. Microbial variations may also impact the function of the microbiome by increasing intestinal permeability while impairing the absorption of nutrition, food metabolization and immune system regulation [3]. An altered intestinal microflora and its complications can influence the pathology of various diseases, including aging.

A distinct microbial composition in the GIT has been linked to aging and age-associated inflammation. For example, a decrease in the anti-inflammatory bacterial species was found in aged mice, including Faecalibacterium prauznitzii and Bifidobacteria spp. [4]. Research also showed a sudden reduction in the Firmicutes phyla and an increase in the Bacteriodetes phyla to occur from adulthood to old age, resulting in a decreased Firmicutes to Bacteriodetes (F/B) ratio [5]. As the two most dominant phyla in the gut microbiome representing up to 90%, the F/B ratio is critical for the production of short chain fatty acids (SCFAs) such as butyrate and propionate [6]. Overall, age-related dysbiosis can further the progression of aging, inflammation and frailty, causing a negative impact on systemic health and longevity.

The role of nutrition in regulating a healthy gut constitution is paramount. A recent comparison of adults, elders and centenarians living in longevity villages and urbanized towns was conducted in South Korea to characterize the role of diet patterns and the living environment in healthy aging [7]. Significant differences in gut microbiota were found. For example, the abundance of Firmicutes was higher in the longevity village residents, while proportions of Bacteroidetes were elevated in the urbanized elders. A greater abundance of Lactobacillus was also found in the subjects from longevity villages. Moreover, microbial analyses of centenarians—individuals with an exceptionally low incidence of chronic illness, reduced morbidity and extended healthy lifespan—revealed higher bacterial diversity than elders and adults from the same region. The presence of immunological and metabolic health bacterial species was richer in centenarians than in other groups, including Proteobacteria, Actinobacteria and Verrucomicrobia, Akkermansia, Christensenellaceae and Lactobacillus. These results were consistent with previous analyses of gut-microbial signatures in centenarians [8]. Bacteroides are also increased in centenarians, potentially from a diet rich in soy protein and isoflavones [7]. However, proportions of anti-inflammatory Faecalibacterium and Prevotella were consistently reported lower in centenarians than the elderly and adults, which was also observed in subjects of urbanized towns. Reported dietary habits of the centenarians in longevity villages revealed an intake of three diverse meals per day, with a healthy appetite reported. Moreover, centenarians exhibit anti-inflammatory properties and an increased expression of pro-inflammatory markers (e.g., IL-18, TGF-β1) that help fight against chronic low-grade inflammation associated with aging [9]. Overall, current data on centenarians suggest that healthy aging and a healthy gut environment is influenced by diet and the environment.

## 3. Cellular Senescence as a Central Dogma of Human Aging

As discovered by Hayflick and Moorhead in 1961, cellular senescence is an intrinsic aging process wherein cells undergo irreversible growth arrest due to their inherent finite lifespan or exposure to stress [10]. The number of senescent cells increases exponentially with age in various tissues and organs, playing a critical role in the development and pathogenesis of age-related diseases. Notably, cellular senescence is known as one of the major hallmarks of aging [11]. As an inherent anti-cancer mechanism, senescent cells lose the ability to proliferate and perform apoptosis in response to DNA damage [12]. However, these cells remain highly metabolically active within the tissue environment and exhibit a hypersecretory phenotype known as the (SASP). The SASP constitutes several proinflammatory cytokines and molecules that work to mediate the paracrine activities of senescent cells [13]. Proinflammatory cytokines, chemokines, proteases, lipids, growth factors and insoluble proteins or extracellular matrix (ECM) components make up the SASP [14]. More specifically, the SASP includes tumor necrosis factor-alpha (TNF-α); interferon-gamma (IFN-γ); interleukin (IL)-1α, IL-1 beta (β), IL-6, IL-8 and others; chemokine ligands (CXCL) 1 (CXCL1) and 2 (CXCL2); fibronectin and collagen of the ECM; matrix metalloproteinase-(MMP-) 3, MMP-9 and MMP-12; cluster of differentiation (CD)4+ T cells; and nonprotein secretions such as reactive oxygen species (ROS) [13,14,15,16,17]. However, the composition and intensity of the SASP varies depending on the senescence-inducing factor, cell type and length that the senescent state has been active [13]. Due to the lack of a single biomarker to detect senescent cells accurately, SASP molecules are among a group of markers simultaneously measured to identify cellular senescence [18]. These widely used markers include the increased expression of cell cycle inhibitors (i.e., coupling cytoplasmic p21 (p21^CIP1^), p16 cyclin-dependent kinase inhibitor 2A (p16^INK4a^)), DNA damage (e.g., p53), senescence associated (SA)-β-galactosidase activity and anti-apoptotic proteins (e.g., B-cell lymphoma-extra large (Bcl-xL), myeloid cell leukemia-1 (MCL-1)) [19,20,21,22,23].

The primary role of the SASP is to recruit immune cells to the site of damage and clear the senescent cells through inflammatory mechanisms. As a result, the SASP factors work transiently in a beneficial manner to perform immune surveillance, promote tissue repair and homeostasis and suppress tumorigenesis [12,14,24,25]. These cells may even have a pro-longevity role when presented acutely, as demonstrated very recently by Attaallah et al. who found a positive correlation between lifespan and the ability to induce senescence in cells that underwent spontaneous DNA damage. Authors further explained the findings by stating that an efficient induction of senescence in compromised cells would avoid a potential sub-population of damaged cells [26]. However, an accumulation of senescent cells with age, or chronic senescence, is accompanied by a high rate of secretion of SASP factors which exceeds the immune system’s clearance capacity [14]. Improper disposal of senescent cells or overactivation of the SASP can result in detrimental effects and increase the risk of disease and mortality [24]. Paracrine senescence may also be induced in healthy neighboring cells following the secretion of SASP factors, further contributing to a state of chronic senescence [27].

### Inducers and Metabolic Activity of Senescent Cells

Cells enter a state of senescence in response to tumor suppressive pathways, namely p16^(INK4a)^/retinoblastoma protein (pRB) and p53/p21 wild-type activating fragment (p21^(WAF1)^) [28]. Several factors contribute to the induction of senescence including intracellular and extracellular signals. Intrinsic stimuli include progressive telomere shortening and dysfunction, oncogenic stress, oxidative stress, chromatin remodeling and mitochondrial dysfunction [12]. External stressors that cause DNA damage can also lead to senescence, such as ultraviolet radiation (UV), ionizing radiation and genotoxic agents.

Metabolic stress acts as both a driver and consequence of cellular senescence. Mitochondrial dysfunction such as a disrupted nicotinamide adenine dinucleotide (NAD^+^)/ nicotinamide adenine dinucleotide hydride (NADH) cytosolic ratio can result in adenosine triphosphate (ATP) depletion and ultimately cell cycle arrest [29]. Damage caused by mitochondrial ROS such as the release of chromatin fragments may also drive senescence and promote SASP factors. A disrupted NAD^+^ metabolism can activate the nuclear factor kappa-light-chain-enhancer of the activated B cells (NF)-κB pathway which has a casual role in promoting senescence and the SASP [29,30]. Moreover, the SASP activates CD38^+^ macrophages which further depletes NAD^+^, as seen in senescence-induced dermal fibroblasts and aged mice models. The NAD^+^ metabolism is linked to age-associated functional decline and diseases, with a progressive decline observed in various tissues [31]. Senescent human diploid fibroblasts present glycolysis via elevated glucose consumption and lactate production [32]. Moreover, a decrease in cytosolic NAD^+^/NADH can induce senescence within human diploid fibroblasts. Once senescent, cells are shown to alter the lipid metabolism, exhibiting high activity of the sphingomyelin-ceramide pathway in fibroblast and endothelial cells, further promoting inflammation [29,33]. Additionally, free polyunsaturated fatty acids (PUFAs) are increased in the membrane of senescent cells and may accumulate as triglycerides in lipid droplets or convert into oxylipins, thereby contributing to the inflammatory arm of the SASP [34]. Hyperglycemia can also promote senescence and subsequent metabolic dysfunction (e.g., decreased sirtuin expression, accumulation of cystolic NADH, activated mitochondrial dysfunction-associated senescence) [29].

## 4. Gut Microbiome and Its Link to Cellular Senescence

### 4.1. Microbial Dysbiosis May Influence a Senescent State

In the early 1900s, the link between cellular senescence and tissue destruction was drawn by Elie Metchnikoff who proposed them to be byproducts of intestinal permeability and subsequent metabolite secretion [35]. Only recently, however, has the literature begun to consider the relationship between senescence and microbial dysbiosis. A groundbreaking study by Saccon et al. directly investigated microbial composition in senescent models [36]. In aged mice, gut microbial signatures were identified in relation to markers of cellular senescence and inflammatory SASP factors. Findings revealed the expression of p16^Ink4a^, p21^Cip1^ and IL-6 to be negatively correlated with the genus Dorea in the ileum. Moreover, IL-1β showed a negative connection to Peptococcaceae, while CXCL1 was positively linked to Staphylococcus and Clostridiales. In the caecum, an abundance of Firmicutes and a reduction in Bacteroides was linked to the expression of CXCL1. Other markers such as p16^Ink4a^ and monocyte chemoattractant protein-1 (MCP-1) showed central connections to several bacterial species in the caecum as well. In the ileum, caecum and colon, however, similar patterns among various species were revealed. For example, a positive correlation between all of the markers mentioned, in addition to TNF-α, was found with Clostridiales, Staphylococcus and Lachnospiraceae. Similarly, Coriobacteriaceae and Akkermansia were negatively correlated with each of the markers in all of the tissues. Through these results, the authors successfully introduced a relationship between cellular senescence and microbial composition, suggesting microbial dysbiosis to play a role in senescence.

### 4.2. Microbial Metabolites Promote SASP Damage

The production of a diverse array of bioactive metabolites and other small molecules is a key component to the function of the gut, acting as the most probable signaling links between the gut microbiota and the host [37,38]. In fact, even a single metabolite and its subsequent effects (e.g., tissues affected, dietary context, systemic levels of the metabolite) can either promote host health or cause toxicity. SCFAs comprise a large class of the small-molecule metabolites produced by the fermentation of dietary fiber in the gut. The key roles of SCFAs include maintaining gut barrier integrity, secreting gut hormones, suppressing inflammation and carcinogenesis, regulating chromatin and more [37,39]. Specifically, butyrate is well known for its role in metabolic regulation and maintaining the integrity of intestinal endothelial cells for gut permeability [40]. The gut microbiota is also responsible for regulating the synthesis and uptake of bile acid [37]. Bile acids, such as deoxycholic acid, have antimicrobial properties and enable the absorption of lipids, cholesterol and fat-soluble vitamins [41]. However, a high concentration of bile acids can result in oxidative stress, DNA damage, mutation and apoptosis. Once within the colon, bile acids are deconjugated by bacterial enzymes (e.g., microbial bile salt hydrolases) and converted into secondary bile acids. Both primary and secondary bile acids modulate the host metabolism through endocrine functions and have important implications for human health [42]. Alterations to the intestinal bile acid pool are linked to several diseases including irritable bowel syndrome, liver cancer and obesity.

Intestinal dysbiosis disturbs the function and synthesis of microbial metabolites. Of note, age-related dysbiosis results in a leakage of proinflammatory microbial products through impaired intestinal permeability [43]. The translocation of proinflammatory microbial products into the bloodstream then occurs, such as the secretion of bacterial lipopolysaccharides (LPS), making way for systemic impact. In turn, various inflammatory molecules are upregulated (e.g., TNF-α, IL-6, IL-1) which promotes a state of chronic inflammation and furthers the SASP phenotype. Dysfunctional immune surveillance also occurs as a byproduct of dysbiosis, thereby impairing the removal of senescent cells.

## 5. Skin Health and Its Relation to Microbiome and Aging

### 5.1. Skin Homeostasis Plays a Role in Healthy Aging

The skin and the gut microbiome draw many comparisons with regards to purpose and function [44]. Both act as the body’s main interfaces with the external environment and must therefore be maintained at homeostasis. The physical barrier as well as commensal microbiota present in the skin and the gut are essential components for proper maintenance [45]. The skin is particularly vulnerable to damage by frequent exposure to environmental factors such as air pollution, tobacco smoke, nutrition and personal care products [46]. As a result, premature aging of the skin can occur which is accompanied by undesired aesthetic indications and impaired skin function. Such disruption to skin health may result in systemic harm. In fact, previous research suggests the skin to be a major source of serum inflammatory markers. Notably, Hu et al. observed a rapid increase in both skin and serum cytokine levels following acute disruption to the epidermal permeability barrier in young- and normal-aged mice [47]. Additionally, a significant reduction in inflammatory markers in the epidermis and serum of aged mice was found upon correction of the epidermal functional abnormalities with topical treatment. The authors concluded that the skin is a likely origin and contributor to age-associated systemic inflammation. More recent research extended the investigation to aged humans with epidermal barrier disruption [48]; treatment with a lipid-based dermatological agent successfully reduced circulating cytokine levels, specifically IL-1β and IL-6, to levels comparable to young controls. An increase in such pro-inflammatory cytokines is associated with chronic aging disorders, including cardiovascular disease, Alzheimer’s disease and diabetes. Therefore, considering the epidermal dysfunction that accompanies age and its apparent relationship with systemic inflammation, the skin may play a role in the pathogenesis of chronic age-related diseases.

### 5.2. Gut-Skin Axis Connects Skin Dysfunction to Metabolic Disruption

Bacterial microbes and their metabolites that enter circulation can travel through the body and affect distant organs and tissues, including the skin [38]. Of note, there is a bidirectional communication pathway between the gut microbiome and the integumentary system, known as the gut-skin axis. In fact, several skin pathologies were shown to present in comorbidity with GI disorders, with a disturbance in the gut microflora associated with inflammatory dermatoses [38,49]. Increased intestinal permeability resulting from dysbiosis has led to an accumulation of bacterial metabolites (e.g., phenols from aromatic amino acids) in the skin and impairment in epidermal differentiation and skin integrity [50,51]. This circulation of metabolites gives rise to an association between cutaneous diseases and metabolic or cardiovascular dysfunction [52]. In particular, a two-way relationship between psoriasis and obesity was found wherein psoriasis predisposes individuals to obesity, and vice versa [53]. Similar alterations to the gut microbiota of psoriatic patients and obese individuals are observed, as well as shared pathophysiology including an increase in microbial byproducts (i.e., adipocytes). Moreover, patients with severe psoriasis have an increased risk of death from cardiovascular disease, malignancies, diabetes, kidney disease and other systemic illnesses [54].

Senescent cells commonly accumulate in the skin, triggering inflammation through the SASP and contributing to several types of skin dysfunction. The unique crosstalk between the gut and the skin presents an opportunity to target skin senescent cells in hopes of resolving skin disruption and associated metabolic disruption, simultaneously. Suppression of the SASP pathway may improve skin health and help restore microbial imbalance, through the gut-skin communicative pathways. Similarly, modulating the gut microbiome directly is a promising approach to treating skin diseases.

### 5.3. Age-Related Skin Microbial Dysbiosis

The skin is also home to a microbial community consisting of millions of bacteria, fungi and viruses, known as the skin microbiome [55]. As seen within the gut, a delicate balance between commensals and pathogens is critical to maintain homeostasis of the skin barrier. A disruption to the skin microbiome is associated with numerous skin diseases. There is significant evidence suggesting age-related microbial changes in the skin, with a recent study revealing differing microbial signatures found in cheek microbiomes of older versus younger Chinese women [56]. For example, Proteobacteria and Actinobacteria phyla were more significantly abundant in the older skin, whereas younger individuals showed a higher prevalence of Bacteroidetes and Firmicutes phyla. The younger skin also showed higher alpha diversity in species’ richness, whereas a predominance of Streptococcus, Lysinibacillus and Bacillus was found in the older age group. Moreover, a difference in transepidermal water loss (TEWL) and sebum production was found between the two groups, which are indicators of skin aging. The authors therefore concluded that an altered skin microbiome can promote skin aging. Another recent analysis was performed regarding the skin microbiome and its association with clinical skin aging parameters including pigmentation, wrinkles and texture [57]. A microbial shift resulting from puberty was demonstrated, as older, post-pubescent groups showed a lack in several taxa. In addition, youth and middle-aged groups demonstrated an enrichment of Staphylococcus, suggesting it to play a role in skin barrier homeostasis. Malassezia was also identified as the most dominant fungi on human skin, specifically for middle-aged and elder individuals. In fact, the authors inferred the skin to be in a state of senescence when an abundance of Malassezia was present. These findings further support an interrelationship between microbial variations, the skin and cellular senescence.

## 6. The Impact of Microbiome and Cellular Senescence on Skin Health and Diseases

Cellular senescence has been discussed in association with a variety of age-related diseases [58]. However, some tissue sites are more commonly populated than others with senescent cells in aged individuals, including the skin [21,59]. The skin is a unique area of discussion with regards to cellular senescence as it is our body’s largest organ and is closely related to microbial dysbiosis, as presented earlier. Its size makes it an attractable target for senescence accumulation and therapy. Moreover, the skin is vulnerable to premature aging and can therefore mediate the onset of premature senescence. Although senescent cells accumulate in the later years of life as a result of aging, frequent skin exposure to stress-inducing factors such as UV radiation (UVR) can accelerate aging phenotypes and induce premature senescence in younger individuals [60,61]. The inflammatory phenotype following a senescent state can potentially contribute to the development of chronic diseases, as previously discussed. It is therefore important to understand the role of senescent cells in the skin and actively protect against their accumulation in order to promote a healthy lifespan.

### 6.1. Consequences of Senescent Cell Accumulation in the Skin

**Figure 2:**
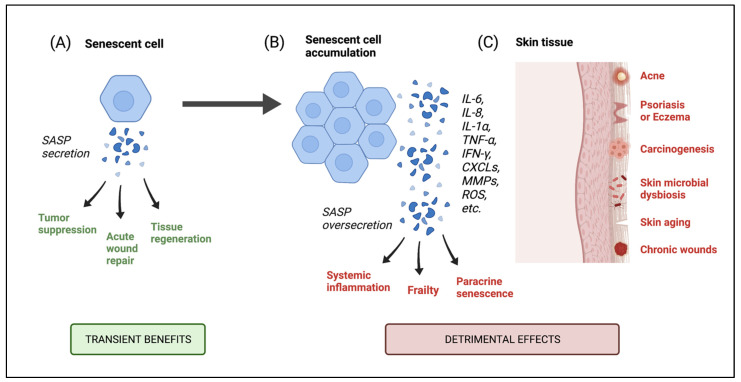
Schematic representation of the effects of cellular senescence on skin tissue. (**A**) A senescent cell secretes senescence associated secretory phenotype (SASP) factors for transient tissue benefits. (**B**) However, an accumulation of senescent cells results in significant harm to the host. An oversecretion of the proinflammatory SASP factors can onset systemic or chronic inflammation, cause frailty and induce senescence to normal neighboring cells via paracrine mechanisms. (**C**) SASP overexpression can also influence the development and pathology of several cutaneous diseases. IL = interleukin; TNF-α = tumor necrosis factor alpha; IFN-γ = interferon gamma; CXCLs = chemokine ligands; MMPs = matrix metalloproteinases; ROS = reactive oxygen species. Created with BioRender.com.

Senescent cells are found to accumulate in skin tissue with age [21,59]. The slow accumulation of these cells can promote chronic inflammation in the skin and persistent degradation of the ECM, leading to several cutaneous pathologies (Figure 2) [24]. As our body’s largest organ and most outer layer, the skin is responsible for protecting against external stimuli and pathogens [62]. The skin’s microenvironment is made up of keratinocytes, melanocytes and fibroblasts, with keratinocyte cells comprising the majority (90–95%) of the epidermal cell population [63,64]. Keratinocytes act as regulators of skin barrier functionality and work to maintain epidermal homeostasis, which is largely dependent on the rate of keratinocyte cell proliferation [62]. However, when senescent, keratinocytes lose the ability to proliferate thereby impairing regenerative function [65]. Keratinocytes are shown to express senescence in vitro and in vivo following UV-B irradiation and subsequent DNA damage [66]. Similarly, dermal fibroblasts are commonly studied in relation to senescence and exhibit an increase in senescence with age, which may interfere with healthy secretion and remodeling of the ECM [21,27,67]. Lastly, senescent melanocytes are shown to limit the proliferation of keratinocytes and contribute to epidermal atrophy [27]. The presence of senescence in various skin cell types contributes to the degradation of skin function seen with age.

Senescent cells are able to spread senescence to surrounding healthy cells via the bystander effect, possibly initiating a systemic impact [68]. ROS released by senescent cells is shown to cause bystander effects through activation of NF-κB [69]. Similarly, SASP factors demonstrate signaling capabilities that promote SASP reinforcement, resulting in paracrine senescence induction in normal cells [70]. An in vivo model of immunocompromised NOD scid gamma (NSG) mice further showed an accumulation of senescent cells to contribute to the bystander effect, demonstrating a high frequency in multiple senescence markers around senescence-induced treatment sites including dermal fibroblasts [71]. Local senescence can therefore have an adverse impact on regional tissue, exacerbating inflammation of the skin in a manner difficult to contain.

### 6.2. Skin Diseases Associated with Senescence and Microbial Implications

The etiologies of various dermatological conditions are linked to inflammation. Given the proinflammatory nature of the SASP markers, the role of senescent cells in the skin was recently investigated for different pathologies. The literature has widely focused on the role of senescence in skin aging; however, the mechanisms of the SASP may be linked to a variety of other skin conditions as well. A greater understanding of associations between cellular senescence and skin disease states is important, considering the relationship between skin homeostasis and gut microbial composition. Recent studies investigated skin pathologies in relation to senescence and associated SASP pathways, suggesting either direct or indirect correlations. Each of the disorders discussed herein contributes to the overall function, health and longevity of the skin and may therefore influence the gut microbiome.

#### 6.2.1. Skin Health and Aging

Skin aging is a shared aesthetic concern world-wide. It is characterized by decreased collagen and gradual epidermal thinning and manifests clinically as wrinkling or sagging, loss of elasticity, laxity and rough-textured appearance [72,73,74]. Many patients seek non-invasive cosmetic procedures for the management or prevention of cutaneous manifestations of aging, with 83.4% of patients motivated by the idea of achieving a more youthful appearance [75]. Thus, factors contributing to tissue aging were of recent discussion, with cellular senescence at the forefront. The critical role that senescent cells play in the development of skin aging phenotypes has become well-established [28,65,74,76]. This can be attributed in part to the overlap of factors involved in intrinsic aging mechanisms and the behavior of senescent cells. The SASP factors secreted by senescent cells can compromise the function and integrity of the skin and contribute to premature aging phenotypes. For example, TNF-α secreted by senescent cells in excision repair cross-complementation group 1 (ERCC1)-deficient mice is shown to promote apoptosis in neighboring fibroblasts, leading to tissue atrophy and subsequent premature skin aging and a shortened lifespan [77].

Increased expression of MMPs is another contributing factor to age-related skin changes. MMPs remodel the ECM by degrading proteins including collagen, fibronectin, elastin and proteoglycans [78]. The function of MMPs is necessary for epidermal differentiation as well as tissue remodeling and repair (e.g., prevention of wound scars) [78,79,80]. However, when senescent cells accumulate, several SASP proteases including MMP-1, MMP-3 and MMP-10 are consistently overexpressed, resulting in an altered ECM microenvironment [14,81]. Senescent models also upregulate additional MMPs; MMP-12, MMP-13 and MMP-14 [14]. A continuous, SASP-mediated degradation of ECM components such as collagen and elastin can significantly impair the dermal response to UVR, leading to photoaging [76,78]. Other skin aging phenotypes such as wrinkle formation, sagging and laxity can result from alterations to collagen and elastin by MMPs [79].

An impaired epidermal barrier resulting from aged skin can contribute to systemic inflammaging, which is also highly associated with age-related alternations to the gut microbiome [82,83]. The gut-skin axis was previously studied in relation to skin aging. Earlier reports suggest oral probiotics to be beneficial in mitigating several indications of skin aging, including acidic skin pH, oxidative stress, photodamage and skin barrier dysfunction [84]. Bacterial species, particularly Lactobacillus and Bifidobacterium, interact with dermal fibroblasts in a photoprotective manner, thereby exhibiting an anti-aging effect [44]. These beneficial bacteria also exhibit anti-inflammatory properties on human intestinal cells for the regulation of tight junctions. Specifically, the expression of pro-inflammatory cytokines, IL-6 and IL-1β, as well as MMPs, was reduced in Caco-2 cells following treatment with an exopolysaccharide derived from Lactobacillus plantarum HY7714. These findings suggest communication between the gut microbiome and the skin for achieving skin longevity.

#### 6.2.2. Skin Carcinogenesis

Age-associated changes to the skin, particularly its immune composition (e.g., reduced Langerhans cells and antigen-specific immunity), result in an increased susceptibility to cancer and infections with age [85]. Senescent cells work transitorily to suppress tumorigenesis by initiating the expression of tumor suppressive proteins, p53 and p16^INK4a^ [86]. However, when accumulated, the same proinflammatory and growth-stimulating SASP molecules are shown to influence the etiology of cancer proliferation, suggesting cellular senescence to be a driving factor behind skin carcinogenesis. Various cell culture models demonstrated premalignant activities and tumor growth mechanisms of senescent fibroblast SASP factors, specifically through functional loss of the tumor suppression protein p53 [87]. Detailed by a new study (2020), an overexpression of p16 in the epidermis demonstrates pro-proliferative and pro-tumorigenic effects, creating a tumor-initiating environment that is supportive of cells carrying an oncogenic mutation [88]. However, the same report revealed that chronic p16 expression failed to constitute a full SASP response as only select SASP markers were upregulated (i.e., CXCL1, CXCL9 and transforming growth factor beta (TGF-β)). Nonetheless, the functional role of p16 in skin tumorigenesis is evident by its ability to promote early premalignant lesions in the skin through paracrine-mediated pathways and may be treated with senolytic therapies for the elimination of p16-expressing cells. These findings are important given that a state of chronic senescence can result in the overexpression of p16. Another recent study, by Alimirah et al., further investigated the relationship between skin carcinoma development and senescence-induced SASP factors in vivo [86]. Findings correlated an upregulation of SASP mediators, p38 mitogen-activated protein kinases (MAPKs) and IL-1α, with a progression in squamous cell skin carcinoma. The induction of senescent cells was shown to promote tumor growth and convert benign tumors to malignant. A subsequent ablation of the senescent cells further validated the promoting mechanisms of SASP, as tumor size, malignancy and p38 MAPK and MAPK/extracellular signal-regulated kinases (ERK) signaling were all reduced following elimination. Moreover, MMPs secreted by senescent fibroblasts are associated with high tumorigenicity as well as the development of squamous cell skin carcinoma [86,89]. These findings highlight the potential of senescence-based therapies for skin carcinoma and other malignancies, given the SASP-associated promoting mechanisms.

Microbial communities of both the gut and the skin are discussed in the current literature with a relation to skin carcinogenesis [90]. Inflammation resulting from a dysbiotic state at either site may promote skin carcinogenesis directly or through indirect mechanisms. Specifically, an alteration of immune pathways (e.g., Th17, regulatory T cells (T-reg)) by the gut results in malignant formation within the digestive tract. Similar carcinogenic effects in the skin may be seen by the microbes inhabiting the skin. Further research may investigate the use of gut and skin microbes as anticancer agents through anti-inflammatory and tumor suppressive mechanisms (e.g., T-reg induction).

#### 6.2.3. Dyspigmentation

The production of melanin in melanocyte skin cells, known as melanogenesis, regulates skin pigmentation. Several inflammatory factors are shown to influence the regulation of melanogenesis, including IL-8, IL-1, IL-6, IFN-γ and TNF-α [91]. Considering these factors fall within the SASP, senescence may play an indirect role in dyspigmentation issues such as hyperpigmentation (e.g., melasma) or hypopigmentation, vitiligo and melanoma susceptibility. Senescent fibroblasts are found to accumulate at age-related pigmentation sites [92]. Recent findings of increased melanin content and ploidy in senescent cells confirm SASP components secreted by UVB-induced senescent fibroblasts to have melanogenesis modulatory abilities [93]. Moreover, skin pigmentation is shown to lighten following the elimination of senescent fibroblasts [92]. These findings support the notion of a cross-talk between melanogenesis regulators and senescent cells. Recent discussion further highlighted the presence of several premature senescence markers in the pathogenesis of melasma and vitiligo [94]. For example, an increased synthesis and production of IL-1β, IL-6 and hepatocyte growth factor (HGF) genes were found in vitiligo fibroblasts when compared against normal control cells [95]. A premature senescence phenotype in dyspigmented skin may give rise to age-related secreted proteins in the skin and subsequently affect melanocyte functionality. Overall, findings suggest that SASP factors influence the development of uneven pigmentation through a potentially harmful relationship.

Recent discussion has highlighted the potential use of microbes to protect against photodamage-induced melanogenesis [96]. Several bacterial species are able to produce products that may prevent altered skin pigmentation. Of note, melanin can be harvested from Streptomyces glaucescens, while serotonin (i.e., another factor largely involved in skin pigmentation) may come from the Escherichia or Enterococcus species. The topical administration of probiotics also demonstrated antioxidative effects against UVB-induced oxidative stress and hyperpigmentation, implying skin microbial modulation to impact pigmentation [97].

#### 6.2.4. Psoriasis and Atopic Dermatitis

Psoriasis is a common and chronic skin disease mediated by T cells and characterized by hyperproliferation and poor differentiation of epidermal keratinocytes; it is also recognized as a systemic inflammatory disorder [98,99]. Research reveals higher levels of SASP-related plasma cytokines and chemokines in psoriatic patients as compared to individuals without psoriasis, including IL-6, IL-1 receptor antagonist (IL-1Ra) and TNF-α [98]. Given its chronic nature, sustained inflammation can occur in psoriasis patients thereby increasing immune activity through T cell populations [98]. Moreover, a higher proportion of senescent T cells was observed in psoriasis patients, with more pronunciation in long-term psoriatic cases (≥15 years) [98]. Such findings imply premature immunosenescence to be associated with psoriasis. The systemic nature of psoriasis has led the discussion on its link to the gut microbiome. In fact, the notion of the microbiome playing a critical role in the immunopathogenesis of psoriasis is well-established. [100] Gut microbial alterations seen in psoriatic patients, such as an elevated F/B ratio and reduced abundance of SCFA-producing bacteria (e.g., Faecalibacterium prausnitzii and Akkermansia muciniphila) along with its common GI comorbidities presented earlier, suggest a significant relationship between gut health and psoriatic disease [101,102].

Atopic dermatitis (AD; also known as eczema) is another leading, chronic skin disease governed by varying inflammatory and immunological pathways. The inflammatory cascade of AD involves several cytokines including IL-4, IL-13 and IL-1, some of which are linked to the SASP [103]. T cell signaling factors (i.e., type II helper T cells (Th2), type I helper T cells (Th1)) are also implicated in the initiation and maintenance of atopic dermatitis. Its pathogenesis can be largely attributed to skin barrier dysfunction characterized by inhibited keratinocyte differentiation and diminished tight junctions, which are also traits seen in senescent cells [65,104,105]. Low-grade inflammation resulting from accumulated senescent cells may contribute to the chronicity of AD given the inflammatory loop driven by cytokines and Th2 cells [104]. However, studies identifying the abundance of senescent cells and their secreted markers in eczema diseased models are needed before conclusions are met. Distinct microbial patterns are observed in patients with AD compared to healthy controls with a notable decrease in the abundance of Bifidobacterium with a lower concentration correlated with AD disease severity [106]. Decreased microbial diversity and a reduction in anti-inflammatory bacterial species (e.g., Bacteroides fragilis and Streptococcus salivarius) are also associated with consecutive AD and eczematic lesions, respectively. Meanwhile, infant AD patients exhibit increased Akkermansia muciniphila and Clostridum and may be less likely to develop AD when a diverse gut microbiota is present at 1 week old [106,107]. The strong association between AD and the microbiome has led some authors to conclude that clinicians should consider the state of the gut microbiome when managing a patient with AD [107].

#### 6.2.5. Acne Vulgaris

Acne vulgaris is a chronic and recurrent skin condition with a multifactorial etiology [108]. Many individuals, especially females, experience an initial or continuous onset of acne during adulthood. Its pathogenesis has been in part linked to local immune reactions and the abundance of bacterial species Propionibacterium acnes (P. acnes), leading to non-inflammatory (e.g., comedones) or inflammatory (e.g., papules, nodules) skin lesions. Research shows that the colonization of P. acnes may trigger a release of proinflammatory cytokines IL-8 and IL-12 and exogenous proteases which may enhance the transcription of IL-1α, TNF-α and MMPs [109]. Acne seen in adult patients may be therefore exacerbated by the presence of senescent cells, given the parallel inflammatory molecules secreted by the SASP.

However, some evidence has interestingly pointed to an association between acne and healthy aging. For example, telomeres of longer length were found in the white blood cells of individuals who previously suffered from acne, suggesting a protective mechanism against premature skin aging [110]. Such a finding may help explain the delay in skin aging phenotypes (e.g., epidermal thinning, wrinkle formation) exhibited by acne sufferers. Contrarily, the p53 pathway was shown as upregulated in acne patients, which is a common indicator of senescence. Additionally, serum levels of pro-inflammatory cytokines including IL-1β, IL-8 and IL-12 are also associated with acne [111]. Thus, more studies are needed to decipher the beneficial or harmful role of acne in aging and better understand its relationship to cellular senescence and SASP molecules.

The relationship between the gut microbiome and acne vulagris was well investigated over the past century. Specifically, the role of diet in the intestinal microbiota has been highlighted with regards to acne. Dietary factors (e.g., red meat, dairy, high glycemic index) were suggested to influence the development of acne through increased levels of IGF-1 and insulin [112]. As discussed earlier, a dysbiotic gut can result in the secretion of microbial metabolites, including IGF-1. Additionally, the release of metabolites through the mTOR pathway may then disturb the gut microbiota, initiating a positive feedback loop. Through such mechanisms, the state of acne may be aggravated by the intestinal flora.

#### 6.2.6. Chronic Wounds

Although acute senescent cells have a beneficial role in tissue repair and regeneration, ongoing senescence may underly the development of chronic wounds. Chronic wounds develop from acute wounds when the healing process is incomplete or interrupted by various factors including excess levels of inflammatory cytokines [113]. This results in a perpetual non-healing state and poor outcomes. It is well known that senescent fibroblasts are prevalent in chronic wounds such as venous and pressure skin ulcers, suggesting senescence to play a critical role in impaired healing likely due to the over-secretion of inflammatory SASP factors [114,115]. Moreover, a recent study discovered a link between cellular senescence and diabetic skin, demonstrating a higher abundance of senescent cells in aged and diabetic wounds [116]. Wilkinson et al. also found the prolonged expression of SASP inflammatory chemokines, CXCL1 and CXCL2 to harm wound repair pathology. In fact, a selective CXCL2 antagonist was able to reverse delayed wound closure and accelerate skin healing in mice and human models. Another recent study found an accumulation of senescent cells and SASP in radiation ulcers in both human and animal ulcer models following radiotherapy, suggesting cell senescence to play a role in the development of radiation ulcers [117]. Senescent cells in radiation ulcers also induced senescence and inflammation to adjacent cells, thereby furthering the accumulation. Given that each stage of wound repair requires cellular signaling from chemokines and other regulators, the adverse impact of senescence and its associated mechanisms on chronic wounds is evident [118].

The chronicity of wounds is largely characterized by the colonization of pathogenic bacteria at the site of the wound and a subsequent formation of an adhesive and protective matrix, known as a biofilm [119,120]. In fact, bacterial biofilms are present in over 90% of chronic wounds while in only 6% of acute wounds [121]. Specifically, Staphylcoccus aureus and Pseudomonas aeruginosa are identified as common biofilm generators and are frequently found in chronic wounds, alongside Peptoniphilus, Enterobacter, Stenotrophomonas, Finegoldia and Serratia species [119,120]. The bacterial load associated with chronic wounds contributes to wound healing delays, suggesting the microbiome to play a significant role.

#### 6.2.7. Skin Immunity

The decline of the immune system is considered to be a hallmark of aging [122]. More specifically, the gradual deterioration of the immune system that accompanies aging, otherwise known as immunosenescence, results in a reduced capacity of the immune system, frailty and an accumulation of senescent cells [67]. A state of immunosenescence often leads to chronic, low-grade systemic inflammation, termed inflammaging [67,85]. The accumulation of senescent cells is one contributor to inflammaging due to the inflammatory mediators of the SASP which considerably alter the skin microenvironment [64]. Resident immune cells that comprise the microenvironment include Langerhans cells, dendritic cells, macrophages, monocytes and T cells. Aged individuals exhibit T cell functional dysregulation (e.g., CD4+/CD8+ ratio reduction), characterized by a loss in CD28, which initiates the production of TNF-α and IFN-γ and subsequently contributes to systemic inflammaging [64,67,123]. These changes may be explained by fibroblast senescence in aged persons or impaired epidermal barrier function. Skin immunity may also be impacted following increased expression of IL-6 from the SASP. IL-6 is critical in the regulation of the immune response and is implicated in the induction and pathogenesis of various autoimmune diseases in the very elderly [124]. However, the relationship between skin immunity degradation and premature senescence has not yet been investigated. Studies designed for this specific purpose would be beneficial in understanding how and when immunosenescence occurs in younger individuals.

The gut microbiome has profound effects on innate immunity and impacts many systemic immune factors [125]. Age-related intestinal dysbiosis is associated with immune dysregulation; impaired intestinal epithelial integrity causes transmucosal leakage of bacterial antigens (e.g., SCFA, neurotransmitters) into systemic circulation, leading to immunosenescence and the development of autoimmune disorders [126]. One mechanism by which the gut microbiota can impact immunity is through the induction of Th17 cells [127]. Th17 cells come from pro-inflammatory T helper cells and are characterized by their production of cytokines including IL-17, IL-22 and IL-21 [128]. Moreover, Th17 cells are known to be uniquely pathogenic in chronic inflammatory and autoimmune diseases, including those of the skin (e.g., psoriasis). In vivo experiments demonstrate an induction of Th17 cells in the host upon microbial adhesion to intestinal epithelial cells [127]. In particular, twenty bacterial strains were identified with adhesive characteristics that may lead to the induction and accumulation of Th17 cells. The diverse list of species includes Clostridium, Bifidobacterium, Ruminococcus and Bacteroides, among others. Considering the imperative role of T cells in skin immunity, gut-mediated cytokine expression such as from Th17 cells may exacerbate the SASP and progress immune dysfunction in the skin.

## 7. Therapeutic Strategies for Targeting Cellular Senescence

With emerging evidence of the role that cellular senescence plays in age-related pathologies, the demand for novel anti-senescence therapies is high. Two distinct approaches to target senescence and its implications were identified in the recent years: the use of senolytic agents wherein senescent cells are selectively induced to perform apoptosis and the use of senostatic treatments which interfere with SASP signaling pathways to inhibit proinflammatory functions [122]. Several pharmaceutical agents were identified to have anti-senescence abilities, especially in combination with plant polyphenols. However, certain drugs are associated with serious side effects (Table 1). Thus, nutraceutical compounds were proposed as an alternative treatment modality. Moreover, probiotics exhibit pro-longevity and anti-inflammatory properties that must be discussed further with anti-senescence consideration.

### 7.1. Pharmaceutical Agents and Their Drawbacks

#### 7.1.1. Senolytics

Dasatinib (D) is an anticancer agent and tyrosine kinase inhibitor that has been leading in research as an effective senolytic agent. It was investigated in combination with quercetin, a polyphenol-rich plant flavonol, as a combinatorial therapy (D+Q). Several studies demonstrated the benefits of D+Q therapy on alleviating senescent cells. For example, when used to treat radiation ulcers of human skin fibroblasts and mice following radiotherapy, D+Q successfully cleared senescent cells through apoptotic pathways [117]. The senolytic therapy worked to mitigate the radiation-induced skin ulcers by decreasing swelling and DNA damage caused by irradiation and promoting proliferation of the epithelial tissue for repair. Other recent findings showcase the ability of D+Q to reduce the number of senescent cells in individuals with idiopathic pulmonary fibrosis, a senescence-associated fatal disease [136]. As the first in-human clinical trial to investigate senolytics, Justice et al. established initial evidence that short-term administration of a senolytic treatment can improve physical dysfunction [136]. To follow, Hickinson et al. investigated the ability of senolytics to decrease senescence burden directly following a three-day oral administration of D+Q to humans with diabetic kidney disease [137]. Findings showed a significant reduction in senescent cells eleven days after treatment, as well as decreased levels of SASP-mediators (i.e., p16 and p21) in the skin and reduced circulating SASP factors (i.e., IL-1α, IL-6, MMP-9, MMP-12) in blood. Early findings of this “hit-and-run” treatment approach with senolytics demonstrate great potential; however, the new senolytic drug class is accompanied by unknown side effects. Hematological toxicity is commonly associated with the continuous administration of D, contributing to its well-established side effect profile; however, its combinatory effect with Q has yet to be determined [129]. As the ongoing clinical trial of Hickinson et al. continued into Phase Two, the use of D+Q senolytic therapy remains cautionary until further conclusions are drawn. A recently published risk-benefit analysis discussed the need to address several factors in upcoming research including validation of its clinical benefits in humans, characterization of the risk profile specific to senolytic-use and a consensus on treatment protocol [129]. Although there was only one serious adverse event from the concluded clinical trials, the small participant pool (twenty-three subjects in total) likely made for a favorable outcome [129,136,137]. Larger longitudinal clinical trials will provide a greater assessment of potential risk from D+Q therapy. Navitoclax is another anticancer drug that was identified as a senolytic given its dual inhibition of Bcl-xL and Bcl-2, anti-apoptotic proteins [138]. However, the non-selective nature of navitoclax often causes severe systemic implications, such as thrombocytopenia, through the off-target reduction in non-senescent cell types following long-term oral administration [130]. A recent technology named proteolysis-targeting chimera (PROTAC) demonstrated the ability to reduce platelet toxicity by converting navitoclax into a Bcl-xL-specific compound that is less toxic to platelets [131]. Contrarily, an overexpression of Bcl-xL was shown to contribute to longevity for successful aging in centenarians and decreased premature senescence, as compared to septuagenarians (i.e., normal aged individuals) [139]. Thus, the role of Bcl-xL must be investigated further to validate the use of Bcl-xL inhibitors as senolytic therapy.

The use of chimeric antigen receptor (CAR) T cells was discovered recently as a potential, more personalized alternative to current drugs. These cells are engineered to act as a synthetic receptor to target antigens of interest and are lately considered a significant advancement in personalized cancer therapy [140]. Given its target specificity, recent investigations focused on extending the clinical use of CAR T cells to address senescence-specific antigens [141]. CAR T cells were able to remove senescent cells in cell culture and in vivo models by targeting a cell-surface protein (i.e., uPAR) that is upregulated during senescence [141]. As the therapeutic potential of novel agents such as CAR T cells continues to emerge, clinical trials must take place to establish safety profiles in hopes of developing improved senolytics.

#### 7.1.2. Senostatics

Rapamycin is an anticancer and immunosuppressive compound that effectively interferes with the secretion of SASP factors [142]. By inhibiting the mammalian target of rapamycin (mTOR), a key regulator and promoter of SASP, rapamycin suppresses the translation of cytokine IL-1α and subsequently diminishes NF-κB transcriptional activity [143]. Currently, rapamycin stands as the only pharmacological intervention to extend the lifespan in all studied models, from yeast to mammals [144]. Recent discussion has suggested rapamycin to be most beneficial when used pre-disease as a preventative measure rather than as treatment following the onset of aging disorders [133]. Further support of rapamycin therapy led some to equate the dangers of not taking rapamycin to those of smoking for elders, given data on its ability to increase the lifespan in older and middle-aged mice (by 9–14% and 60%, respectively) [133,145,146]. However, research is needed on the safety of long-term use of rapamycin due to potential metabolic side effects (i.e., glucose intolerance, insulin resistance, hyperglycemia), although such harmful effects may be mitigated by intermittent administration or low daily dosing and medical observation [133]. The literature also provides strong evidence of metformin as a senostatic agent through demonstrated interference with the NF-κB pathway and thus suppression of SASP proinflammatory signaling [147]. Metformin is able to attenuate the release of SASP cytokines including IL-6, IL-8 and IL-1β through immunomodulation, reduce oxidative stress and even perform anti-apoptotic and senolytic activities [147,148]. Discussion on the anti-aging benefits of metformin led to ongoing clinical trials that are investigating its impact on frailty in older adults with prediabetes [149]. Such research intends to extend the clinical use of metformin beyond diabetes. However, a very recent study brings into question the safety of metformin treatment in older individuals without diabetes [134]. Contrary to its pro-longevity effect on younger models, metformin shortened the lifespan of non-diabetic, aged C. elegans by exacerbating age-associated mitochondrial dysfunction and resulting in a lethal response. Similar to rapamycin, metformin suppresses the senescent phenotype in a reversible manner thus suggesting that continuous treatment may be required to achieve efficacy with these agents [150]. In order to assess long-term treatment, ongoing trials are needed that measure efficacy, toxicity and other side effects of metformin in individuals of varying ages and disease populations to validate use as an anti-aging drug.

### 7.2. Nutraceuticals and Diet as a Novel Approach to Fighting Senescence

The substantial side effects of senolytic and senostatic pharmaceutical agents warrant the need for alternative therapies. Recently, the use of nutraceutical compounds to target cellular senescence was considered [151]. Quercetin is a bioactive flavonol that is found in a variety of fruits and vegetables [152]. The biological and pharmacological importance of quercetin is evident by its potent antioxidant, anti-inflammatory and immunomodulatory properties. Moreover, it is an effective anticarcinogenic agent known to induce apoptosis, making it a first-generation senolytic agent [15,152]. Quercetin effectively kills senescent cells by inhibiting phosphoinositide 3-kinase (PI3K), a mediating pathway for survival regulation and growth factors [65,153]. Inhibition of PI3K was recently validated by a study that demonstrated senescence reversal in human dermal fibroblasts following inhibition of 3-phosphoinositide dependent protein kinase 1 (PDK1), a molecular kinase that belongs to PI3K [154,155]. Successful senescence eradication resulted in proliferative recovery and restored skin regeneration capacity [154]. When combined with Dasatinib, or D+Q therapy, the burden of senescent cells significantly decreased, which is well-established in the literature as discussed previously. However, anti-aging benefits are also exhibited when treatment involves quercetin as a sole agent; topical treatment using quercetin can increase the cellular lifespan of senescent human fibroblasts for a rejuvenating effect [156]. However, as a natural polyphenol, quercetin faces poor bioavailability, solubility and chemical instability limitations. Recent research sought to overcome such drawbacks by mediating the senolytic action of quercetin with nanoparticles. Lewinska et al. synthesized quercetin surface functionalized Fe_3_O_4_ nanoparticles (named MNPQ) and demonstrated senolytic and senostatic benefits in human foreskin fibroblasts [157]. Additionally, photoprotective agents may be considered to treat senescence since UVR-induced damage and subsequent ROS overproduction is a leading initiator of senescence [158]. Treatment of UV irradiated human skin with quercetin recently showed great protection against photoaging through the suppression of SASP factors (i.e., MMP-1, COX-2, collagen degradation) and interference with senescence-associated transcription pathways (i.e., JAK2, AP-1, NF-κB) [159]. These findings suggest a greater opportunity of quercetin as a senotherapeutic apart from dasatinib. Further research is needed to understand quercetin in different senescence-related contexts for the development of novel phytocompound-based therapies.

Other phenolic compounds demonstrated anti-senescence benefits including oleuropein aglycone (OLE), a derivative of the olive oil plant, and epigallocatechin gallate (EGCG) found in green tea leaves [151]. OLE has reduced senescence markers and SASP factors in vitro, but direct investigation on its impact on the lifespan is lacking, as well as in vivo studies. Contrarily, EGCG was studied in vivo using catechin-fed mice, demonstrating an increased lifespan and decreased systemic SASP factors. The benefits of green tea catechins are currently exercised in oral supplements and cosmetic products and can be extended to senolytic and senostatic therapies following further validation. Quite similar to quercetin, fisetin is a highly active nutraceutical that is considered one of the pioneering senolytic agents. Its anti-senescence properties can be explained in part by its chemical makeup of hydroxyl groups which allows for free radical scavenging activity and subsequent antioxidative benefits [160]. The literature on the benefits of fisetin for targeting senescence is well-established, with recent findings identifying it as the most potent senolytic among ten flavonoids tested, including quercetin, EGCG, catechin and others of high interest [161]. Results showed selectively reduced senescence and associated markers in murine and human tissue, as well as health and lifespan extension in progeroid and aged mice following an intermittent dietary intake of fisetin. Given the putative role of fisetin as compared to the studied counterparts, as well as its established safety profile, supplementation may be a great option for treating senescence. Ongoing clinical trials to evaluate fisetin may bring its researched properties one step closer to therapeutic translation.

Considering the efficacy of the combination of D+Q, a nutraceutical-based combinatorial approach to targeting senescence may be another solution. A recent study showed that a combination of nutraceutical supplements significantly increased the length of whole and short telomeres in mid-age (forty to fifty-five years old) healthy adults [162]. These findings are the first to reveal that telomere length can be influenced by antioxidants from a daily blend of vitamins (i.e., vitamins of B-, D-, E- and K-groups), omega 3-6-9, L-glutamine and probiotics (i.e., kefir, Bifidobacterium bifidium, Lactobacillus casei, Lactobacillus reuteri, Lactobacillus acidophilus), suggesting a new therapeutic avenue for targeting senescence-related telomere dysfunction.

### 7.3. Probiotics and Prebiotics as New Therapeutics for Managing Aging

Probiotics are live organisms that elicit health benefits when administered in adequate amounts [163]. There is very recent discussion on the use of probiotics as a potential senotherapeutic agent. The well-established relationship between gut microbiota and healthy aging suggests probiotic bacteria to be potential modulators of cellular senescence [164,165]. A variety of bacterial strains are shown to influence dermatological conditions or their associated pathways (Table 2), which can be attributed to the established anti-inflammatory and anti-oxidative properties of probiotics. Probiotics also demonstrated great pro-longevity benefits [165]. New literature explored the benefits of probiotics on senescence, with a recent study (2020) demonstrating the probiotic Lactobacillus fermentum (L. fermentum) to alter mTOR signaling and suppress the activation of SASP factors in senescence-induced murine preadipocyte cells [166]. The mitigation of the deleterious effects of the senescent cells was possibly a result of secretory metabolites by L. fermentum beneficial gut modulation, according to the investigators. A later investigation measured the direct effect of D+Q senolytic treatment on gut microbial composition, revealing the ability of the senolytic therapy to alleviate intestinal senescent cell abundance, SASP factors and inflammation through gut immunomodulation [36]. The mRNA expression of senescent cell markers p16 and p21 and SASP inflammatory markers (i.e., CXCL1, IL-1β, IL-6, MCP-1 and TNF-α) were significantly lower in all of the intestinal segments of the aged mice treated with D+Q, compared to placebo-treated aged mice. Moreover, a lower abundance of Firmicutes was found in the ileum and GIT of the treated aged mice. Lactobacillus, Proteobacteria and Sutterella were also reduced following D+Q treatment, while Akkermansia was found to be higher in abundance. Overall, findings showed that the effect of D+Q senolytic treatment had the most effect on the ileum microbiota compared to cecal, colon or fecal microbiota. Although the presented findings are promising, the differences in gut composition did not reach statistical significance, likely due to the small sample size and high standard deviation. Thus, further research is needed to assess gut modulation by D+Q treatment.

The amalgamation of probiotic bacteria with plant polyphenols was also proposed as a synbiotic treatment for senescence [164]. This novel approach may improve the limited bioavailability of polyphenols alone through interactions with probiotic bacteria in the gut. Recent studies demonstrated the promising effects of combining probiotics with bioactive, phenol-based agents that act as prebiotics. In particular, a synbiotic formula made of the ayurvedic poly-phenol rich herb Triphala and a probiotic blend including Lactobacillus plantarum, Bifidobacteria longum spp. infantis and L. fermentum showed pro-longevity abilities by significantly extending the lifespan of Drosophila and reducing age-related physiological stress, oxidative stress and low-grade inflammation [165]. Aged Drosophila were treated over the course of thirty days with either the synbiotic formula, the probiotic blend, L. fermentum alone or Triphala alone. Treatment with the probiotic blend proved to be effective; however, the synbiotic formula demonstrated a greater impact in every regard. Notably, supplementation with the synbiotic resulted in a greater increase in longevity (twenty-six days; 60%) compared to probiotic treatment alone (twenty-four days; 55%). L. fermentum and Triphala alone also increased the lifespan, although with less impact (sixteen days and fourteen days, respectively). The synbiotic and probiotic also led to significant reductions in the total weight of Drosophila and rescued total glucose elevation at day thirty, compared to controls. Interestingly, all groups reduced total triglyceride levels; however, L. fermentum and the probiotic formula were able to reduce the elevation to the level of day zero completely. SCFA production, specifically butyrate, was substantially increased by the synbiotic treatment as well. The improvement in the lifespan and metabolic markers shows great promise for the use of probiotics in aging. A synergistic effect from combining prebiotics with probiotics may also be achieved by compounds targeted specifically for cellular senescence.

A diet rich in SCFA-producing dietary fibers may additionally help age-related microbial dysbiosis and, in turn, suppression of the senescent phenotype. For example, supplementation with butyrate demonstrated the ability to counterbalance age-related microbiota dysbiosis [40]. A study showed that a high-fiber diet with 5% inulin for a duration of 4 weeks resulted in an altered gut microbiome in aged mice and increased levels of butyrate, acetate and total SCFA production. Notably, a significant increase in β-diversity was observed, along with a reduction in Ruminococcus spp. and Rikenellaceae. The models also showed lower gene expression of inflammatory markers including IL-1β, TNF-α and IL-6 and exhibited an increased anti-inflammatory microglial profile compared to mice fed on a low-fiber diet. These findings support the intake of high fermentable, prebiotic fiber in resolving age-related dysbiosis.

## 8. Use of Metabolomics in Targeting Senescence

The human metabolome represents the thousands of small-molecule metabolites that make up the human body [182]. Targeted metabolic profiling can be used for the absolute quantification of known structured metabolites (i.e., endogenous or exogenous) with high accuracy. Similarly, untargeted metabolome analyses measure global, detectable metabolites in the body, allowing for wide clinical applications. Use of metabolomics along with the other omics technologies (e.g., genomic, transcriptomic, proteomic) is a powerful tool for measuring metabolic changes in biofluids, tissues and organs [183]. Its application can be leveraged to unravel the integrated mechanisms of aging [182]. High-throughput methods show that increased metabolites and low abundance molecules are associated with aging, while metabolites associated with amino acids, lipids, carbohydrates and redox metabolism may act as biomarkers of aging and longevity [182,184]. A recent analysis further revealed a hub of metabolites (i.e., NAD^+^, NADPH, alpha-ketoglutaric acid (αKG) and beta hydroxybutyrate (βHB)) to be central mediators of aging, defining them as the aging metabolome [185]. NAD^+^ specifically was found to govern the SASP through enhanced glycolysis and mitochondrial respiration, making it a central factor to cellular senescence [186].

Metabolomics can also be used to assess metabolites retained in the skin. Metabolites can be found in excretions from skin sweat glands, sebum production, protein degradation and interstitial fluid. Skin tissue can also harbor intestinal microbiota metabolites originated from a dysbiotic microbiome and permeable intestinal barrier [51]. The role of skin metabolites in systemic diseases, such as SASP factors, makes skin metabolomics a valuable tool for identifying characteristic biomarker profiles [187]. Moreover, the accessibility of the skin allows for sampling techniques of various forms: skin biopsy, suction blistering, tape strip, transdermal patch and hydrogel, among others. Nuclear magnetic resonance (NMR) spectroscopy is a recent technique that enables personalized analyses through metabolic fingerprinting. Personalized metabolic profiling can help mitigate interpersonal variations commonly seen in the skin metabolome and improve patient outcomes by understanding the specific needs of an individual.

Although metabolomics is used to understand the role of biomarkers and metabolic products in a variety of skin conditions (e.g., melanoma, psoriasis, atopic dermatitis), senescent skin cells have not yet been investigated in this regard, to our knowledge. The metabolic activity of senescent cells and their secretory phenotype make cellular senescence a great candidate for metabolic profiling. Metabolomic techniques can be used to help identify and quantify metabolites present under the senescent status. Given the lack of a single biomarker to detect senescence accurately, the use of metabolomics would be beneficial in refining the impact of the biomarkers involved. Discovery of a senescent metabolome would enable early prediction of chronic senescence, preventing further accumulation of senescent cells and subsequent damage. Identification of influential metabolites can also aid in the development of senescent-targeted therapeutics. For example, systemic toxicity seen with long-term use of pharmaceutical agents may be mitigated with topical treatment to the skin that addresses specific metabolites [65]. Additionally, metabolic pathways underlying senescence or the SASP revealed by metabolomic analyses can be used to formulate anti-senescent therapies that treat the resulting damage while preserving positive effects of senescent cells. Changes to the gut metabolome following probiotic or prebiotic consumption can also be measured with metabolomics to understand its relationship with systemic metabolism better [183]. For example, previous metabolic analyses of adult and aged mice urine and fecal samples using NMR revealed a reduction in age-related metabolic dysfunction (i.e., homocysteine concentration, NAD metabolism) after supplementation with Lactobacillus acidophilus La5 and Bifidobacterium lactis Bb12 [188]. Similar methods can be applied to understand the effect of bacterial therapies in senescent models. Overall, metabolomics can be used to clarify mechanisms of cellular senescence, enabling the design of novel, precision therapeutics.

## 9. Healthy Aging and Skin Health Product Development Opportunities

With a rise in the geriatric population and aging concerns comes the need for novel longevity solutions. Since 2018, the market for anti-senescence therapies has experienced a rapid growth that is expected to reach an all-time high of 644.4 million United States dollars (USD) by 2023 [189]. This rise can be largely attributed to the increase in clinical trials and Food and Drug Administration (FDA) approval underway for longevity and senolytic agents such as D+Q. As so, the global demand for anti-aging solutions is expected to be worth 83.2 billion USD by the year 2027 [190]. Similarly, the use of probiotics is expected to grow at a rate of 10.43% by 2027 which will be largely comprised of products for chronic disease indications [191]. The skincare market is also projected to reach 200.25 billion USD by 2026, with an emphasis on achieving skin health using natural compounds [192]. Several ailments are projected to hold large shares of the future market including UVR damage, wrinkle formation and dyspigmentation.

In considering the anticipated markets for anti-senescence therapies, probiotics and natural skincare, there is a great opportunity to develop novel solutions for skin senescence. The current literature provides a foundation for the design and translation of oral probiotics and prebiotics for senescence-associated pathways. The skin, moreover, is a common site for senescent cell accumulation and a large target in size and therapeutic potential for pathologies that may be triggered through cellular damage to the skin [59]. However, current investigational drugs under review lack topical administration. Thus, a dermatological approach may be used to treat localized regions or indications of senescent accumulation, enabling greater targeted efficacy and bioavailability. Certain industry players have seized this market potential, however more efforts are needed to translate emerging research into consumer products for the treatment of cellular senescence.

## 10. Future Implications

The gut microbiome specifically impacts systemic metabolic pathways, conferring and controlling skin health and healthy aging. This role seems simple, however it is a complex process that involves bidirectional communication. Despite the detrimental effects of senescent cell accumulation, a modulated secretion of SASP factors can enhance the positive influence of senescent cells such as tissue regeneration, reprogramming of the skin microenvironment and accelerated cutaneous wound healing [25]. Therefore, it is important to develop therapeutic strategies that optimize the benefits of senescent cells while minimizing the negative effects. Although current senolytic and senostatic drugs show great efficacy, they are faced with limitations as anti-aging treatments [18]; their poor safety profiles warrant the need for novel, improved anti-senescence therapies. Senolytic therapies have not yet shown efficacy in all senescent cell types which introduces the need for more tissue-specific therapies. Efforts to address senescent- and SASP-related skin indications in an individualized manner would allow for the selective elimination of senescent cells within a single tissue and region. The current literature suggests that cellular senescence influences a range of skin diseases, impairs longevity and influences systemic disruption. However, many factors remain unknown.

Of note, discussion on microbial composition in relation to cellular senescence has only just begun. Further analyses of the gut-skin axis are warranted, such as the presence of bacterial metabolites in the skin of senescent models. Studies observing the change in gut composition in senescence-induced models would also be beneficial. Additionally, microbial analyses of individuals with premature senescence compared to naturally aged persons (e.g., centenarians) would be of value. Upon a greater understanding of the gut microbiota’s role in senescence, preclinical and clinical trials can be performed to investigate the therapeutic potential of select probiotics and prebiotics. Studies can evaluate gut modulation and the systemic response to pharmaceutical senolytics and senostatic agents compared against probiotics and nutraceutical compounds. Knowledge on mechanisms of action underlying cellular senescence is also needed. Such findings can be used to develop novel anti-senescent treatments that are safe and effective, including synbiotic compounds.

Studies can be later extended to investigate the microbiome of the skin in the presence of chronic senescence and perhaps the impact of topical compounds. The use of topical administration would enable localized treatment (e.g., to a chronic wound), thereby reducing the risk of non-senescent cell targeting and preventing systemic toxicity [65]. Moreover, the use of topical senescent therapies could benefit senescence-induced systemic conditions by decreasing inflammation in the skin and improving barrier dysfunction. However, more research is needed on skin senescent models in order to clarify whether anti-senescence therapies impact skin pathologies and if topical agents demonstrate equal or superior benefits. In sum, the intersection of cellular senescence, skin and probiotic bacteria is a promising field of research that requires attention.

## 11. Conclusions

Understanding the relationship between the gut microbiome and healthy aging is fundamental to achieving systemic longevity. Cellular senescence—a major hallmark of aging—is a promising area of research that requires investigation with relation to microbial dysbiosis. As an inevitable, age-related process, cellular senescence can cause severe damage to the host upon accumulation, largely due to overexpression of the SASP and associated metabolic dysregulation. Data from recent findings suggest an intricate relationship to exist between the gut microbiome, cellular senescence and skin health. This proposed relationship is anchored by the SASP and largely influences the aging phenotype and associated diseases. The skin is vulnerable to the accumulation of senescent cells due to its external exposures (e.g., UVR). Recent literature reviewed in this article suggests senescence to partake in numerous cutaneous diseases, all of which compromise function of the skin and general health. The link between skin homeostasis and healthy aging is further supported by recent evidence demonstrating systemic detrimental effects from chronic senescence in the skin, likely through paracrine signaling. Moreover, the presence of bacterial metabolites in the skin due to the gut-skin crosstalk can disrupt skin health, one way being further aggravation of the SASP. Recent investigation has drawn correlations between gut composition and cellular senescence and revealed a distinct microbial composition in the gut in response to senolytic treatment. However, additional studies are needed to advance knowledge on microbial composition and function in the presence of accumulated senescence. Metabolomics is one approach that can help characterize metabolites found systemically and in the skin in order to quantify the impact of specific metabolic activity on senescence.

Additionally, there is a great need for novel anti-senescent therapeutic agents due to the dangerous side effects associated with long-term use of currently available senolytic and senostatic drugs. The impact of diet and environment on cellular senescence has also been discussed, leading to the proposal of probiotic- and prebiotic-based therapies. However, the efficacy of bacterial and nutraceutical compounds, as well as their synergistic effects when combined, must be assessed in relation to cellular senescence. Omics techniques may be utilized to measure changes to the gut microbiota and circulating metabolites following consumption of such agents. Topical anti-senescent treatment is also considered for a more localized approach, given the accumulation of senescent cells found in the skin. Furthermore, the skin is a great target for ameliorating senescence to prevent not only skin disorders but associated systemic dysfunction as well. Research can also be conducted to determine the impact of senescence in younger individuals as a preventative measure. Finally, the role of SASP activation and secretion in different diseased models must be further clarified in order to advance knowledge on host physiology following senescent cell accumulation. Such efforts will aid in the development of anti-aging strategies to improve skin, systemic health and overall longevity for healthy aging.

## Figures and Tables

**Table 1:** Current pharmaceutical agents for the treatment of senescence and their associated adverse effects with chronic, long-term use.

Pharmaceutical Agent	Mechanism	Long-Term Side Effects
Dasatinib + Quercetin	Senolytic	Currently unknown with further investigation required [129].
Navitoclax	Senolytic	Platelet toxicity or thrombocytopenia [130,131]; neutrophil toxicity [132].
Rapamycin	Senostatic	Glucose intolerance; insulin resistance; hyperglycemia; starvation pseudo-diabetes; stomatitis; mucositis; interstitial pneumonitis [133].
Metformin	Senostatic	Shortened lifespan; mitochondrial dysfunction; lethal ATP exhaustion [134,135].

ATP = adenoside triphosphate.

**Table 2:** The role of SASP in various skin indications and evidence of probiotic impact.

Skin Disease	SASP Factor(s) Involved	Probiotic Influence on Factor(s) and Disease
Aging	Upregulated: MMPs [74,167,168] TNF-α [169] IL-6, -8, -1β [169]	Tyndallized Lactobacillus acidophilus was shown to suppress MMPs for wrinkle prevention in photoaged skin through inhibition of elastase activity [169,170]. An enrichment of Cyanobacteria can accompany a decrease in UV-induced damage and pigmentation, suggesting it to be a photoprotective species [57].
Carcinogenesis	Upregulated: p38 MAPK [86] IL-1α [86] p16 ^INK4a^ [88]	Propionibacterium acnes produces conjugated linoleic acid which was shown to inhibit carcinogenesis and modulate the immune system [171].
Dyspigmentation	Modulated: IL-1α, -1β [172,173] TNF-α [174] IL-6 [174] HGF	Tyndallized Lactobacillus acidophilus exhibits antimelanogenesis effects by inhibiting the cAMP pathway and suppressing melanin secretion [170].
Psoriasis and Atopic Dermatitis	Upregulated: IL-6, IL-1 [98,103] TNF-α [98] CXCL1, CXCL2 and CXCL8 [98] T cells [103,104]	Lactobacillus pentosus was shown to decrease levels of TNF-α and IL-6 among other cytokines in psoriasis-like skin [99].
Acne Vulgaris	Upregulated: IL-1β, -8, -12 [111] p53 pathway [110]	Streptococcus salivarius can inhibit the growth of Propionibacterium acnes and downregulate IL-8 in epithelial cells and keratinocytes [175,176].
Chronic Wounds	Upregulated: CXCL1, CXCL2 [177] p16^INK4a^, p53 [178] MMPs [179]	Staphylococcus epidermis can suppress skin inflammation during wound repair [180].
Immunity Decline	Dysregulated: CD4+/CD8+ ratio [64,123] Upregulated: TNF-α [67] IFN-γ [67] IL-6 [124]	Colonization with Staphylococcus epidermis can enhance skin barrier and remodel skin immunity by inducing IL-17A+ CD8+ T cells [181].

MMP = matrix metalloproteinase; TNF-α = tumor necrosis factor-alpha; IL = interleukin; UV = ultraviolet; MAPK = mitogen-activated protein kinases; p16^INK4a^ = cyclin-dependent kinase inhibitor 2A; HGF = hepatocyte growth factor; CXCL = chemokine ligand; CD = cluster of differentiation; IFN-γ = interferon gamma.

## Data Availability

No new data were created or analyzed in this study. Data sharing is not applicable to this article.
